# Dietary curcumin supplementation promotes browning and energy expenditure in postnatal overfed rats

**DOI:** 10.1186/s12986-021-00625-5

**Published:** 2021-10-30

**Authors:** Xiaolei Zhu, Susu Du, Qinhui Yan, Cuiting Min, Nan Zhou, Wei Zhou, Xiaonan Li

**Affiliations:** 1 Department of Child Health Care, Children’s Hospital of Nanjing Medical University, 72 Guangzhou Road, Nanjing, 210008 Jiangsu Province People’s Republic of China; 2 Institute of Pediatric Research, Nanjing Medical University, Nanjing, 210029 Jiangsu Province People’s Republic of China

**Keywords:** Obesity, Postnatal overfeeding, Curcumin, Browning of white adipose tissue, Energy metabolism

## Abstract

**Background:**

Early postnatal overfeeding could result in metabolic imprinting that decreases energy expenditure following white adipose tissue (WAT) gain throughout life. This research investigated whether curcumin (CUR) supplementation could promote WAT browning and activate thermogenesis in postnatal overfed rats.

**Methods and results:**

This study adjusted the size of litters to three (small litters, SL) or ten (normal litters, NL) to mimic early postnatal overfeeding or normal feeding from postnatal day 3. From postnatal week 3 (weaning period), SL rats were fed a standard diet (SL) or a diet supplemented with 1% (SL_1% CUR_) or 2% (SL_2% CUR_) CUR for ten weeks. At postnatal week 13, SL rats with 1% or 2% CUR supplementation had lower body weight and less WAT gain and had an increased lean mass ratio, and their glucose tolerance and blood lipid levels had recovered to normal when compared to SL rats that did not receive the supplement. Moreover, the increased heat generation were consistent with the expression levels of uncoupling protein 1 (UCP1) and other browning-related genes in the subcutaneous adipose tissue (SAT) of the SL_2% CUR_ rats but not in the SL_1% CUR_ rats. In addition, 2% CUR dietary supplementation enhanced the serum norepinephrine levels in SL rats, with upregulated mRNA levels of β3-adrenergic receptor (β3-AR) in SAT.

**Conclusion:**

Dietary CUR supplementation attenuates body fat gain and metabolic disorders in SL, which might be induced by promoting browning of SAT and energy expenditure. Moreover, the benefits were more obvious in SL with 2% CUR supplementation.

**Supplementary Information:**

The online version contains supplementary material available at 10.1186/s12986-021-00625-5.

## Background

Obesity is a chronic metabolic disease owing to long-term energy metabolism imbalance and has become increasingly prevalent [1]. Numerous studies on different nutrition exposures across species have shown that obesogenic factors in the early postnatal period could cause energy metabolism disturbance in the subsequent life process [2], but the mechanism remains to be elucidated. Artificial increase or decrease of the pups in a litter is an ideal method to simulate neonatal over- or poor-feeding, respectively [3]. Studies have shown that the breast milk intake of small litters (SL) (three to four pups) is increased compared with that of normal litters (NL) (eight to twelve pups). Additionally, the triglyceride (TG) content of breast milk from SL mothers was significantly richer than that of the NL group [4, 5].

Our previous study indicated that lower energy expenditure levels occurred in SL rearing (3 pups) rats and that this situation could persist until adulthood [6]. Therefore, on the basis of developmental plasticity, exploring the possibility of regaining energy metabolism balance and discovering its mechanism could provide potential clues for pursuing solutions for obesity caused by early overfeeding.

Adipose tissue is among the critical organs that regulate homeostasis of whole-body energy metabolism [7]. White adipose tissue (WAT) is the primary organ that stores excess energy intake, while brown adipose tissue (BAT) activates nonshivering thermogenesis in which energy is dissipated as heat [8]. As one of the principles to treat obesity is to cause an increase in energy expenditure, activating BAT and increasing its heat production is considered a potential treatment for obesity [9, 10]. In fact, BAT in adults is absent. Fortunately, a third type of adipocytes, named beige adipocytes, have been discovered in WAT. It has been confirmed that beige adipocytes express uncoupling protein 1 (UCP1), a thermogenic marker, similar to BAT [11]. In addition, peroxisome proliferator-activated receptor γ coactivator-1α (PGC1α), a critical regulatory factor of energy metabolism and mitochondrial biosynthesis, is highly expressed in brown and beige adipocytes [8]. Moreover, PGC1α acts as a cofactor that combines peroxisome proliferator-activated receptors γ (PPARγ) with positive regulatory domain containing 16 (PRDM16) to increase UCP1 levels in WAT [12–14]. Many endogenous and exogenous factors, such as sympathetic stimulation, cold exposure, pharmacological and nutritional factors, have been proven to be effective in triggering WAT browning [15]. Therefore, identification of these factors is of great significance in designing strategies for the prevention and treatment of obesity.

Curcumin (CUR), derived from turmeric, is a natural flavonoid component and a safe food additive [16]. Several studies have shown that CUR possesses protective effects against obesity and its related metabolic disorders such as insulin resistance, hyperlipidemia and fatty liver [16]. CUR supplementation can prevent high-fat diet (HFD) induced obesity and pharmacologically administration induced insulin resistance in rats [17, 18]. Recently, function of CUR in inducing WAT browning has gained attention. In vitro studies have found that CUR is able to induce the expression of brown-specific markers in 3T3-L1 adipocytes and primary white adipocytes [19, 20]. Moreover, CUR-treated mice exhibit lower body weight and less fat mass, formation of beige adipocytes, and higher expression of thermogenic genes in inguinal WAT [21]. These studies indicated that CUR might be targeted to control the obesity induced by WAT browning.

It is well known that the early postnatal period is one of the critical stages of WAT development. Our previous study has confirmed that postnatal overfeeding could suppress the expression of browning markers in WAT, reduce heat production and promote lipid accumulation in adulthood rats [22]. CUR can prevent obesity and metabolic disorders, but its effects on postnatal overfeeding remain unknown. We hypothesized that adding a CUR supplement to the postweaning diet could promote WAT browning and energy expenditure, thereby preventing obesity and metabolic disorders in postnatal overfed rats induced by SL rearing.

## Methods

### Animals

All trials conducted on rats in this research received approval from the Ethics Committee of Nanjing Medical University (Permit No. 1905046), and all procedures were conducted in accordance with the Guidelines for Use and Care of Laboratory Animals of Nanjing Medical University. Female Sprague–Dawley rats on gestational day 14 were obtained from the Animal Core Facility of Nanjing Medical University (Nanjing, Jiangsu, China) and raised in a standard environment (a 12-h/12-h light/dark cycle, 22 ± 2℃, 40% ~ 60% humidity). Tap water and food were freely available to all rats.

### Experimental design

Artificial SL rearing (three to four rat pups per litter) allows increased breast milk availability and induces postnatal overfeeding, which mimics overnutrition during suckling in humans [3]. For rats, postnatal week 3 (W3) is the weaning period, puberty occurs at W6-8, and adulthood is W9 and afterwards [23].Our previous studies found that metabolic dysfunctions caused by postnatal overfeeding took place early at W3 and persisted to W13-16 [6, 24]. Therefore, this study selected W3 and W13 as the two key experimental time points to explore the effects of postnatal nutritional environments on adult health conditions.

The experimental protocol is shown in Additional file 1: Supplementary Fig. 1. A total of 11 female Sprague–Dawley rats on gestational day 14 were used in this study. 3 days after birth, the litters were adjusted to 3 male pups per dams (*n* = 8 lactating dams), and the group of litter reduction was called the small litter group (SL, *n* = 24). The normal dams were maintained with 10 male pups (*n* = 2 lactating dams), and normal litter pups were called the normal litter group (NL, *n* = 12). The animals for each group were obtained randomly [25, 26]. At W3 (weaning period), six rats in NL and SL groups were sacrificed respectively. At the same time, the rest of NL rats were fed a standard diet (NL group, *n* = 6), and the rest of SL rats were fed either a standard diet (SL group, *n* = 6) or a diet supplemented with 1% (SL_1% CUR_ group, *n* = 6) or 2% CUR (SL_2% CUR_ group, *n* = 6) until W13. CUR was purchased from Oranika Health Products (95% standardized CUR extract, Richmond, British, Canada). We monitored the weights and food intakes of rats weekly at a fixed time point. The animals at W3 and W13 were sacrificed. The specific experiments at each time point are shown in Additional file 1: Fig. S1.

### Magnetic resonance imaging (MRI)

A 7 T Bruker BioSpec 70/20USR scanner was used in this study. Before the MRI scanning, we weighed the rats and then used 5% isoflurane for anesthetic induction and 1–2% isoflurane as the maintenance dose. Anesthetics were mixed in compressed air and delivered by a nasal mask at a speed of 1 L per minute. After judging that they were anesthetized, the rats were stably placed in the head holder of the scanner to ensure that the magnet could position reproducibly. We monitored the respiratory rate of rats and kept it at approximately 60–80 breaths per minute during the experimental period. This study selected axial sections from liver to bladder for the analysis of adipose tissue and used two sets of multislice spin-echo sequences (TR = 3604.5 ms, TE = 33.0 ms) to obtain 35 T2-weighted anatomical axial slices per rat for W3 rats or 70 for W10 rats, with a thickness of 1.50 mm. We set the field of view as 4.77 × 4.50 cm^2^ (W3 rats) or 7.03 × 6.63 cm^2^ (W10 rats) and the matrix size as 256 × 256, with three averages per slice.

Next, we manually looped the target adipose tissue samples of each section of the images and calculated the pixel areas (ImageJ software, National Institutes of Health, USA). The fat surface area per section was multiplied by the intersection distance to yield the corresponding fat volume. Next, total body fat volume (cm^3^) was converted to mass (grams) by multiplying by 0.9196 g/cm^3^, i.e., the density of adipose tissue [27], and the percent of body fat was roughly estimated by (body fat mass/body weight) * 100%. The body weight minus body fat mass was the lean mass. Finally, the lean mass percentage was calculated as (lean mass/body weight) * 100%.

### Intraperitoneal glucose tolerance test (IPGTT)

This experimental scheme followed the protocol designed in a previous study [28]. In brief, the rats were fasted overnight at W3 and W13, followed by intraperitoneal administration of D-glucose (2.0 g/kg body weight). A small drop of rat tail vein blood was collected at 0-, 30-, 60-, 90- and 120-min time points after administration of glucose. The levels of blood glucose were determined using a glucose meter (Accu-Chek, Roche Diagnostics, Mannheim, Germany). Next, we measured the blood glucose levels of rats at 30-, 60-, 90- and 120-min time points following intraperitoneal administration of D-glucose (2.0 g/kg body weight). Then, the area under the curve (AUC) of glucose was calculated.

### Energy expenditure

At W13, rats were housed in the metabolic chamber individually. The whole-body metabolic phenotyping of rats was recorded through an indirect calorimetry and locomotor activity monitoring system (TSE Phenomaster, TSE, Germany), including metabolic rates (oxygen consumption (VO_2_), carbon dioxide production (VCO_2_)) and heat production [29]. We placed rats in metabolic chambers for 72 h for acclimation and then collected data from them every 15 min for an additional 24 h. The parameter values obtained during the final 24 h were used for statistics, and then the ratio of VCO_2_ versus VO_2_ was calculated as the respiratory exchange ratio (RER). Three rats were selected randomly from each group for detection. Water and food were freely available to all rats in each metabolic chamber.

### Serum and tissue collection

Following overnight fasting, rats were weighed and anesthetized with 300 mg of chloral hydrate per kilogram by intraperitoneal administration at W3 and W13. Then, we collected blood samples from the left ventricle and centrifuged them at 2000×*g* and 4 °C for 15 min. The supernatant was collected and stored at −80 °C for subsequent biochemical analyses. The BAT and three main types of WAT (i.e., subcutaneous adipose tissue (SAT), epididymal adipose tissue (EAT) and retroperitoneal adipose tissue (RAT)) were rapidly isolated, rinsed with normal saline and then weighed. Then, 4% paraformaldehyde was used to fix a portion of the SAT for subsequent sectioning and staining, followed by placing the rest into liquid nitrogen for quick freezing and storage at −80 °C for later experiments.

### Serum measurements

The contents of serum aspartate aminotransferase (AST), alanine aminotransferase (ALT), total cholesterol (TC), TG, high-density lipoprotein cholesterol (HDL-C) and low-density lipoprotein cholesterol (LDL-C) were measured using an automatic biochemical analyzer (7100, Hitachi, Japan). Additionally, serum norepinephrine (NE) and fasting insulin were detected by ELISA kits (Rat Norepinephrine ELISA Kit CSB-E07022r, Rat Insulin ELISA Kit CSB-E05070r, CUSABIO, China). The homeostasis model for insulin resistance (HOMA-IR) index was calculated as: Fasting blood glucose (mmol/L) × fasting serum insulin (μU/mL) /22.5.

### Hematoxylin and eosin (H&E) staining

SAT was fixed using 4% paraformaldehyde overnight at room temperature, followed by sectioning after paraffin embedding. H&E staining was performed on the basis of a standard protocol. Four arbitrary fields of view per rat were analyzed by ImageJ to estimate the adipocyte area.

### RNA isolation and reverse transcription quantitative real-time PCR (RT-qPCR)

Each frozen SAT sample was homogenized in TRIzol (TAKARA, Japan) to isolate total RNA following the manufacturer’s instructions. The extracted total RNA was quantified by spectrophotometry at OD260. Agarose gel electrophoresis was used to analyze the integrity of total RNA. According to the manufacturer’s recommendation, this study prepared cDNA by M-MLV reverse transcriptase (TAKARA, Japan) using 1 µg of extracted total RNA. The experiment utilized a Quant Studio 3 real-time PCR instrument (Applied Biosystems, USA) to amplify cDNA by SYBR Green master mix (Vazyme, China). Target gene expression levels were calculated using 2^−ΔΔCt^ [30] and normalized to the average of the housekeeping gene glyceraldehyde-3-phosphate dehydrogenase (GAPDH). The sequences of all primers involved in this study are shown in Table 1 and Additional file 1: Table S1.

**Table 1:** Primer sequences used for mRNA quantification by RT-qPCR

	Forward primer 5’–3’	Reverse primer 5’–3’
UCP1	ACTGTACAGAGCTGGTGACA	TGGTAGAGAGTTGATGAATCTCGT
PGC1α	GTGGATGAAGACGGATTGCC	TTCTGAGTGCTAAGACCGCT
PRDM16	GGACAGTGACAGAGACAAAAGC	CTGTGAATAGAAGGCCGGTA
TMEM26	TTGCCATGGGCTAGAATCCG	TAAAGGCCTGTGCAGCTACC
PPARγ	ATCAGGTTTGGGCGAATG	TTTGGTCAGCGGGAAGGA
β3-AR	TGCTGTTCCTTTGCCTCCAA	TAGCTACGACGAACACTCGA
GAPDH	GGCTCTCTGCTCCTCCCTGTTCTA	CGTCCGATACGGCCAAATCCGT

*UCP1* uncoupling protein 1, *PGC1α* peroxisome proliferator-activated receptor γ coactivator-1α, *PRDM16* positive regulatory domain containing 16, *TMEM26* transmembrane protein 26, *PPARγ* peroxisome proliferator-activated receptor γ, *β3-AR* β3-adrenergic receptor, *GAPDH* glyceraldehyde-3-phosphate dehydrogenase

### Immunohistochemistry

Immunohistochemistry for UCP1 was performed on deparaffinized sections (8 µm thick) of fixed SAT. For antigen retrieval, the sections were submerged in 10 mmol/L citrate buffer (pH 6.0), heated to 100ºC for 20 min, incubated with 3% hydrogen peroxide for 25 min to block the endogenous peroxidase activity, and then incubated with 3% bovine serum albumin at room temperature for 30 min to prevent nonspecific reactions. This step was followed by incubation with anti-UCP1 (Abcam, USA, 1:500) overnight at 4 °C, rinsing with 0.1 mol/L phosphate buffer and incubation with biotinylated goat anti-rabbit antibody (Servicebio, China, 1:200) for 50 min at room temperature. Signal was detected with diaminobenzidine substrate (Servicebio, China), and the nuclei were counterstained with hematoxylin for 3 min. UCP1 positive expression was determined as a brownish-yellow color, and images were taken with Olympus BX51 light microscope (Olympus, Japan). Image pro plus was used to calculate the percentages of positive areas.

### Western blotting

SAT samples were homogenized in precooled RIPA buffer containing a protease inhibitor cocktail. Following centrifugation at 12,000×*g* and 4 °C for 15 min, the supernatant was collected. The concentration of proteins was detected using a BCA protein assay kit (Thermo Fisher Scientific, Rockford, IL, USA) following the manufacturer’s instructions. Equal amounts of total protein were loaded in each lane of a 10% SDS-PAGE gel, separated, and then transferred onto polyvinylidene difluoride membranes (Bio-Rad, USA). The membranes were blocked with 5% dry nonfat milk for 2 h at room temperature, probed with anti-UCP1 (Abcam, USA) or anti-GAPDH (Proteintech, China) antibodies overnight at 4 °C, and washed 5 times with phosphate-buffered saline and 0.1% Tween 20 (PBST) for 6 min each. Next, the membranes were incubated with a horseradish peroxidase-conjugated secondary antibody for 1 h at room temperature and then washed again with PBST (5 washes for 6 min each). The reaction was determined using a chemiluminescence system (ChemiDoc XRS + , Bio-Rad, USA). The band intensity was analyzed by Image Lab (Bio-Rad, USA).

### Statistical analyses

All data were normally distributed and are presented as the mean ± standard error of the mean. Significant differences between groups at W3 were analyzed using Student’s unpaired *t*-test and at W13 using one-way analysis of variance (ANOVA) followed by a post hoc least significant difference (LSD) *t*-test. A *p*-value < 0.05 was considered to be statistically significant.

## Results

### Body weight, food intake and liver enzymes

**Fig. 1:**
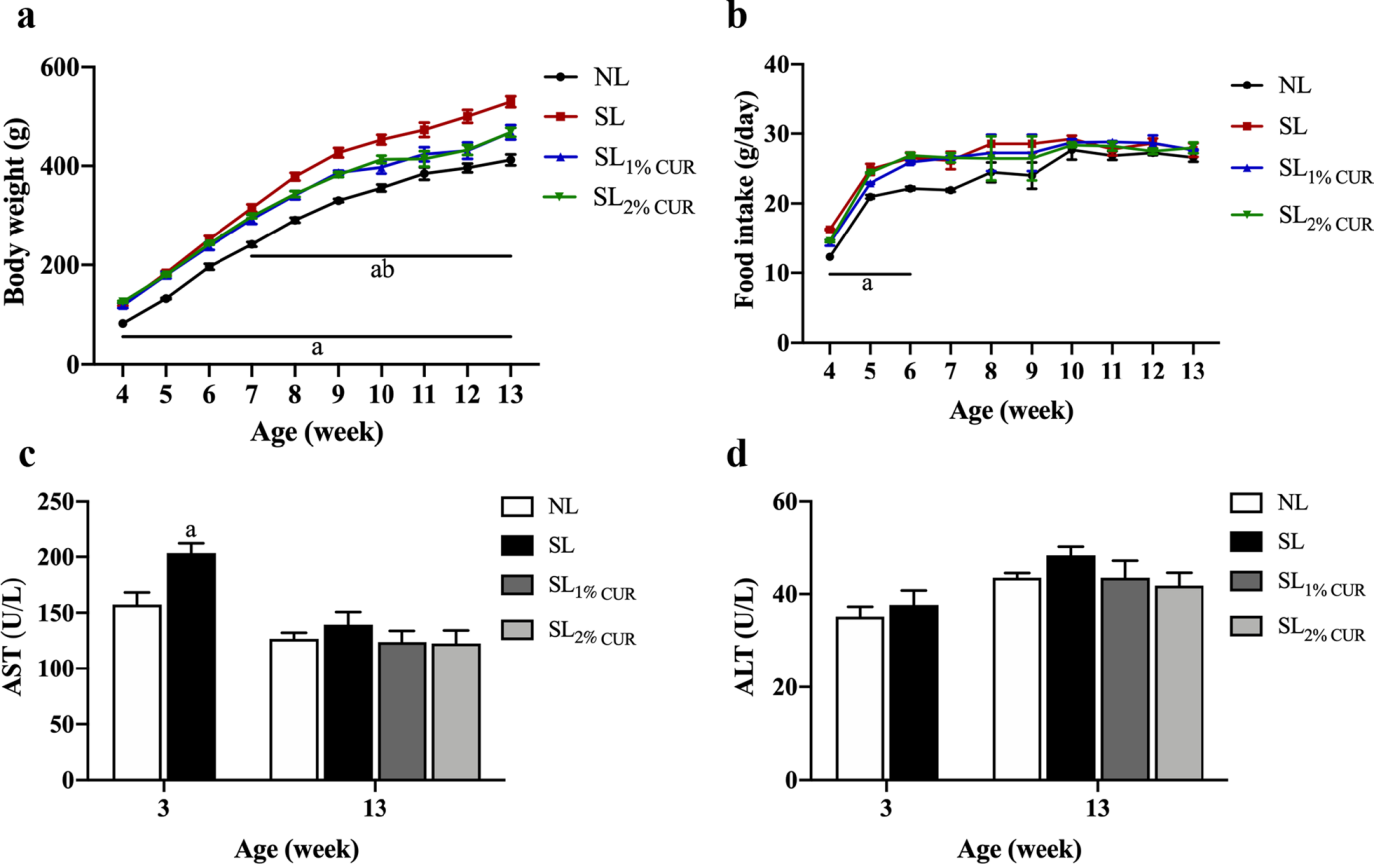
Body weight (**a**) and food intake in rats (**b**) from W4 to W13. Serum levels of aspartate aminotransferase (AST) (**c**) and alanine aminotransferase (ALT) (**d**) in rats at W3 and W13. All values represent means ± SEMs. ^a^*p* < 0.05 versus NL, ^b^*p* < 0.05 versus SL. Body weight and food intake were analyzed by one-way analysis of variance (ANOVA), and AST and ALT levels were analyzed by Student’s unpaired *t*-test at W3 and one-way analysis of variance (ANOVA) at W13. *n* = 6 in each group

As shown in Fig. 1, the weight gain and food intake of rats changed with age in all groups. Compared with NL rats, the body weight of SL rats was significantly higher from postnatal W3 until W13. The weight gained in both SL_1% CUR_ and SL_2% CUR_ rats was lower compared to SL rats after intervention for 4 weeks (Fig. 1a). The food intake of NL and SL rats was basically equal after W6, and the rats fed CUR did not have a difference in food intake in comparison to the SL rats (Fig. 1b).

Next, we determined whether CUR supplementation had a significant effect on the liver function of rats. The SL rats showed a marked increase in their serum AST compared with NL at W3, but no significant difference was observed in serum ALT (Fig. 1c and d). Moreover, the levels of serum AST and ALT in the SL, SL_1% CUR_ and SL_2% CUR_ groups were not different from those in the NL group at W13 (Fig. 1c and d).

### Body composition, adipose tissue mass and size

**Fig. 2:**
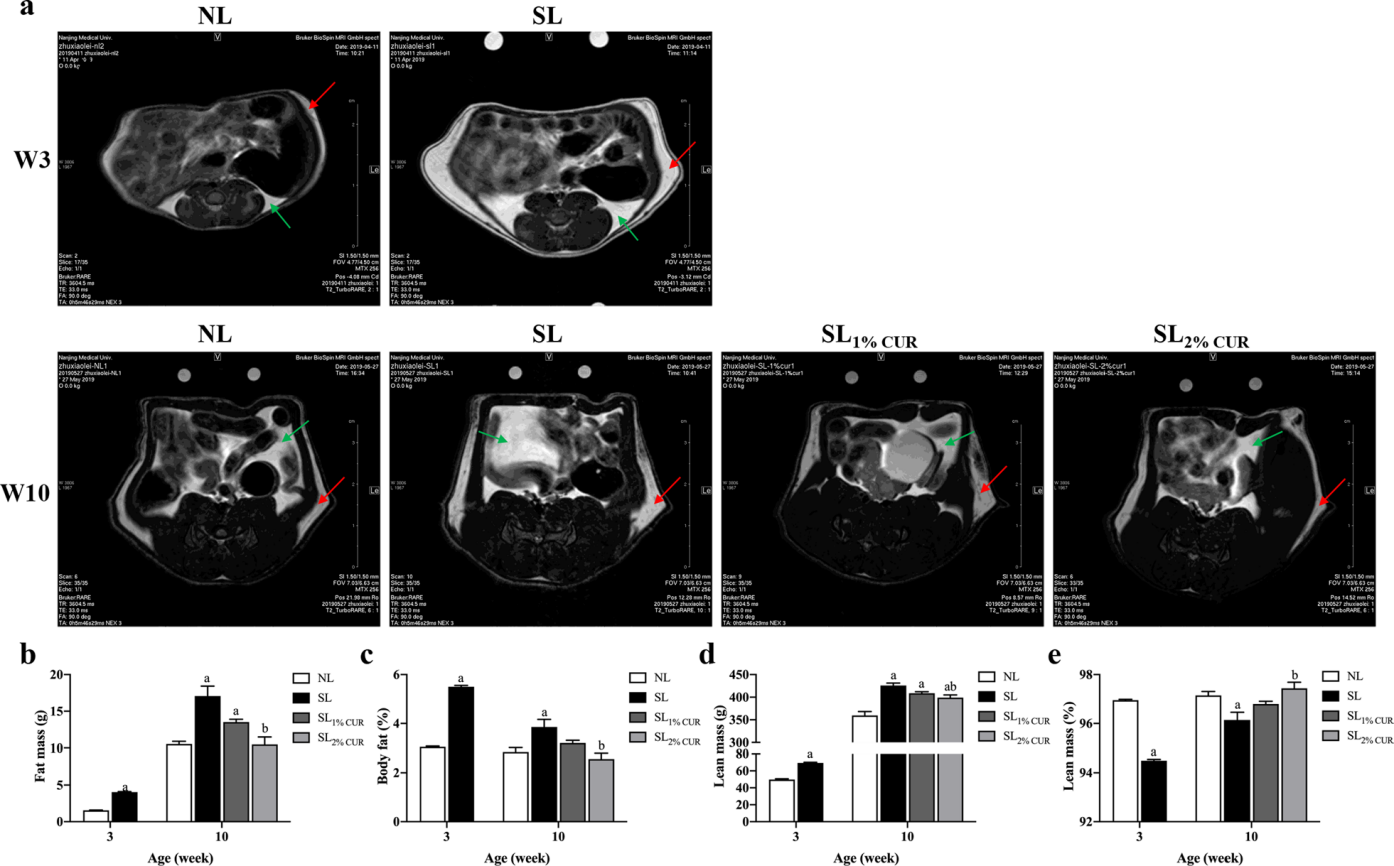
MRI analysis of body fat in rats at W3 and W10 (the red arrow points to subcutaneous adipose tissue, and the green arrow points to visceral adipose tissue) (**a**) followed by calculation of fat mass (**b**), body fat percentage (**c**), lean mass (**d**) and lean percentage (**e**). All values represent means ± SEMs. ^a^*p* < 0.05 versus NL, ^b^*p* < 0.05 versus SL. Statistical analysis was performed using Student’s unpaired *t*-test at W3 and ANOVA at W10. *n* = 3–4 in each group

The differences in body composition between groups at W3 and W10 are shown in Fig. 2. The fat volume and body fat percentage of SL rats were higher than those of NL rats at W3 and W10. Compared to SL rats, SL_1% CUR_ rats had decreased fat volume and body fat percentage at W10 (Fig. 2a). The fat volume and body fat percentage in SL_2% CUR_ rats were significantly lower than those of SL rats and similar to those of NL rats at W10, but this was not true for the SL_1% CUR_ rats (Fig. 2b and c).

The lean mass in SL rats with or without CUR supplementation was greater than that in NL rats at W10, and it was more obvious with the 2% concentration (Fig. 2d). However, the opposite was true for lean percentage, as shown in Fig. 2e.

**Fig. 3:**
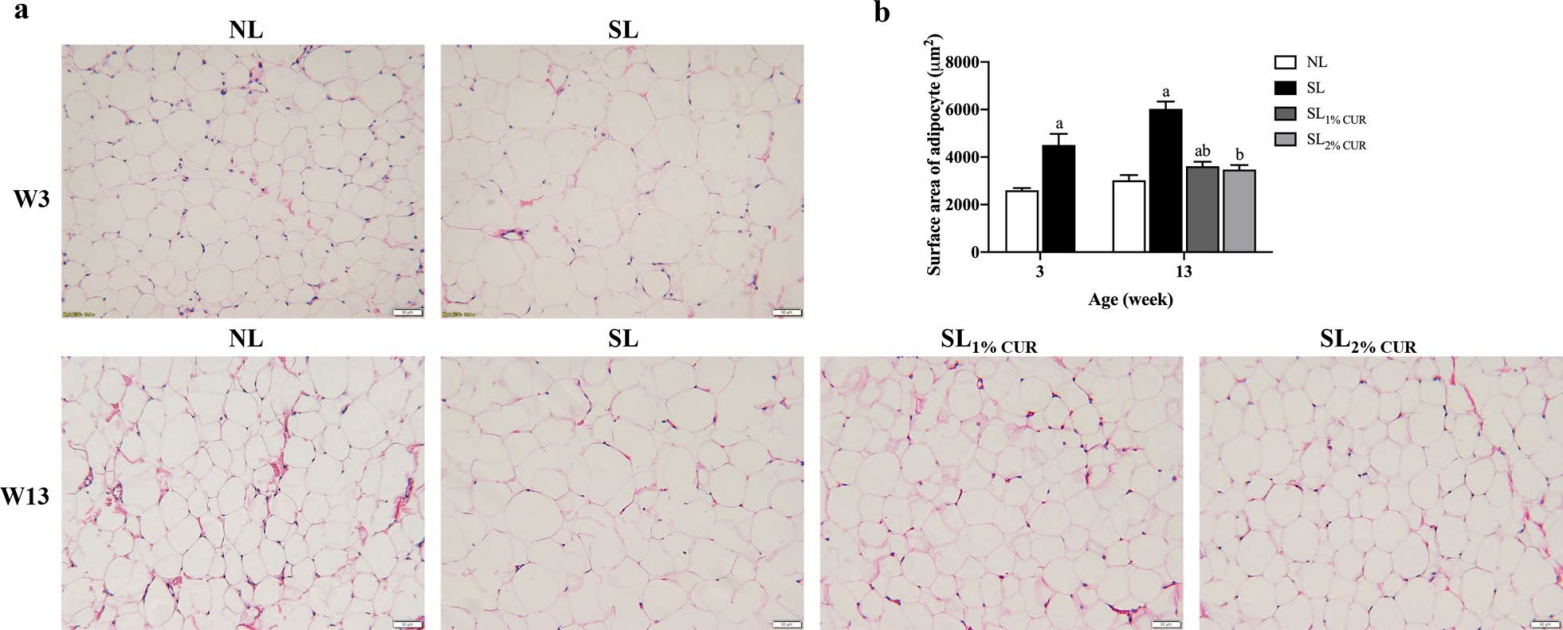
Hematoxylin and eosin (H&E) staining in sections of subcutaneous adipose tissue in rats (200 ×) (**a**) and determination of the surface area of adipocytes (**b**) at W3 and W13. All values represent means ± SEMs. ^a^*p* < 0.05 versus NL, ^b^*p* < 0.05 versus SL. Statistical analysis was performed using Student’s unpaired *t*-test at W3 and ANOVA at W13. *n* = 6 in each group

Similarly, BAT and WAT (i.e., SAT, EAT and RAT) weights in SL rats were higher than those in NL rats at W3 and W13 (Table 2). The results showed that the three main types of WAT weights of SL_1% CUR_ and SL_2% CUR_ rats were lower than those of SL rats, but there was no difference in BAT weight among SL, SL_1% CUR_ and SL_2% CUR_ rats (Table 2). Furthermore, the mass ratio of BAT to WAT in SL rats was lower than that in NL rats at W3 and W13, while that of both SL_1% CUR_ and SL_2% CUR_ rats was close to normal (Table 2). Histologically, the average surface area of subcutaneous white adipocytes in SL rats was larger than that in NL rats at W3 and W13. As expected, the average surface area of subcutaneous white adipocytes in SL rats with CUR intervention (SL_1% CUR_ and SL_2% CUR_) was diminished markedly (Fig. 3a and b).

**Table 2:** Adipose tissue weights in rats at W3 and W13

	NL	SL	SL_1% CUR_	SL_2% CUR_
W3				
BAT (g)	0.11 ± 0.01	0.20 ± 0.02^a^		
SAT (g)	0.56 ± 0.05	1.58 ± 0.04^a^		
EAT (g)	0.08 ± 0.00	0.25 ± 0.03^a^		
RAT (g)	0.08 ± 0.00	0.30 ± 0.03^a^		
BAT/WAT	0.17 ± 0.02	0.09 ± 0.01^a^		
W13				
BAT (g)	0.27 ± 0.00	0.37 ± 0.31^a^	0.31 ± 0.03	0.31 ± 0.31
SAT (g)	4.53 ± 0.17	9.39 ± 1.27^a^	5.53 ± 0.50^ab^	5.74 ± 0.50^ab^
EAT (g)	3.57 ± 0.15	7.20 ± 0.33^a^	4.88 ± 0.30^ab^	4.50 ± 0.42^b^
RAT (g)	2.67 ± 0.18	7.37 ± 0.89^a^	3.88 ± 0.39^ab^	4.77 ± 0.48^ab^
BAT/WAT	0.028 ± 0.002	0.0160 ± 0.002^a^	0.024 ± 0.003^b^	0.022 ± 0.002^b^

All values represent means ± SEMs

Student’s unpaired *t*-test at W3 and one-way analysis of variance (ANOVA) at W13 were performed. *n* = 6 in each group

*W3* week 3, *W13* week 13, *NL* normal litter, *SL* small litter,* CUR* curcumin, *BAT* brown adipose tissue, *SAT* subcutaneous adipose tissue, *EAT* epididymal adipose tissue, *RAT* retroperitoneal adipose tissue

^a^*p* < 0.05 versus NL

^b^*p* < 0.05 versus SL

### Glucose homeostasis and serum lipids

**Fig. 4:**
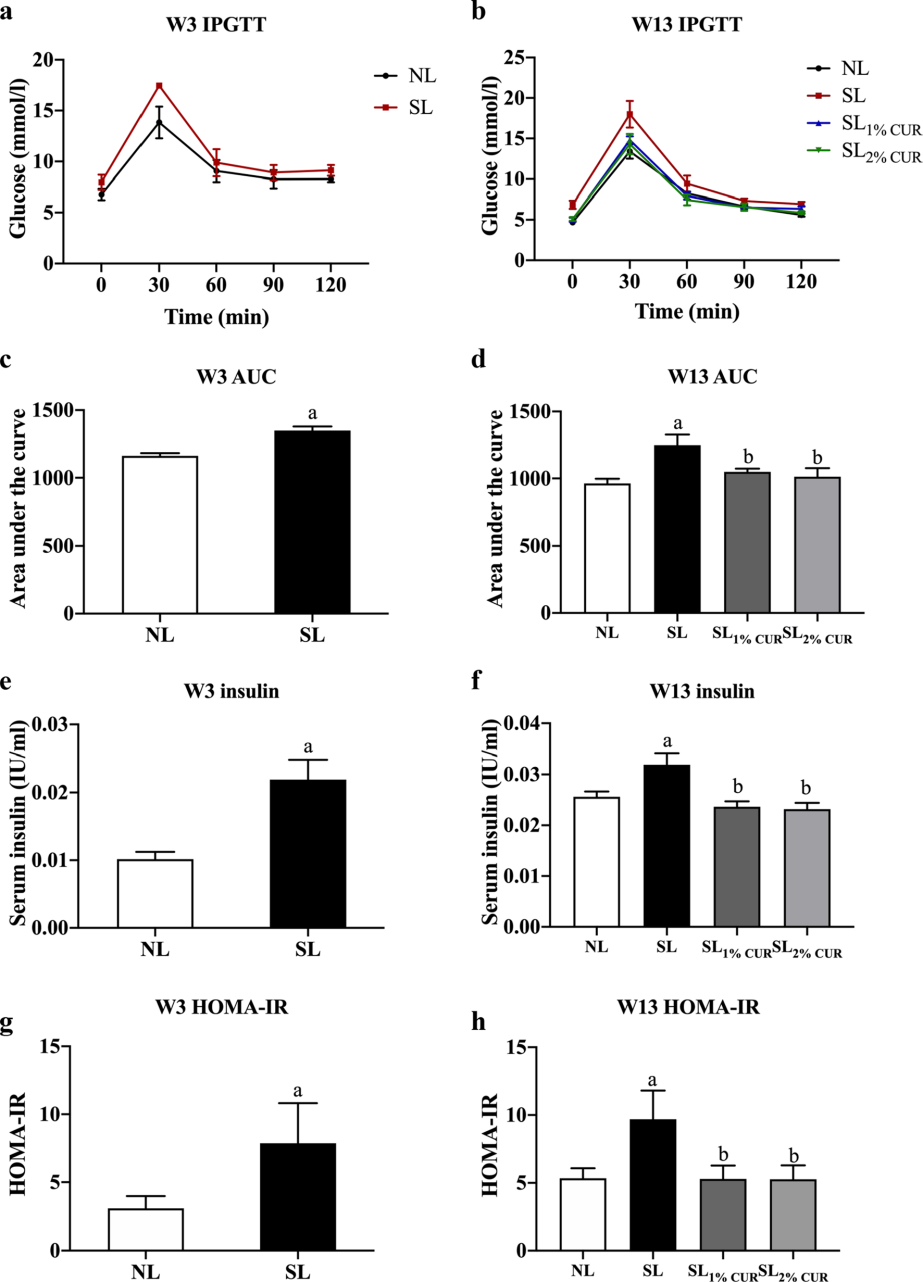
Intraperitoneal glucose tolerance test (IPGTT) (**a** and **b**), area under the curve (AUC) (**c** and **d**), serum levels of insulin (**e** and **f**) and HOMA-IR (**g** and **h**) in rats at W3 and W13. All values represent means ± SEMs. ^a^*p* < 0.05 versus NL, ^b^*p* < 0.05 versus SL. Statistical analysis was performed using Student’s unpaired *t*-test at W3 and ANOVA at W13. *n* = 6 in each group

An IPGTT was conducted in this study to infer the insulin sensitivity of rats. The results showed that the AUC for blood glucose of SL rats was larger than that of NL rats at weaning (W3) and adulthood (W13), suggesting that SL rearing could impair the glucose tolerance of rats. The AUC of the SL_1% CUR_ and SL_2% CUR_ rats was decreased in comparison to SL rats and was similar to NL at W13 (Fig. 4a–d). Consistently, the serum insulin and HOMA-IR of SL rats were higher than those of NL rats, while both indexes recovered to the normal level in the SL_1% CUR_ and SL_2% CUR_ rats (Fig. 4e–h).

Moreover, compared with NL rats, serum HDL-C levels of SL rats were decreased at W3, and there were no obvious differences in serum TC, TG, and LDL-C levels between NL and SL rats (Table 3). Notably, the serum TC, TG, and LDL-C levels of SL rats were all evidently higher than those of NL rats at W13, while those of both the SL_1% CUR_ and SL_2% CUR_ rats were reduced in comparison to the SL rats and close to normal levels. There were no differences in the level of serum HDL-C among the groups at W13 (Table 3).

**Table 3:** Serum lipid biochemical parameters in rats at W3 and W13

	NL	SL	SL_1% CUR_	SL_2% CUR_
W3				
TC (mg/dl)	2.14 ± 0.09	2.00 ± 0.15		
TG (mmol/L)	0.59 ± 0.13	0.64 ± 0.13		
HDL-C (mmol/L)	0.59 ± 0.01	0.46 ± 0.05^a^		
LDL-C (mmol/L)	0.52 ± 0.04	0.55 ± 0.30		
W13				
TC (mg/dl)	1.59 ± 0.07	1.85 ± 0.07^a^	1.58 ± 0.06^b^	1.47 ± 0.06^b^
TG (mmol/L)	0.59 ± 0.06	1.23 ± 0.13^a^	0.60 ± 0.12^b^	0.57 ± 0.04^b^
HDL-C (mmol/L)	0.38 ± 0.02	0.36 ± 0.03	0.34 ± 0.03	0.37 ± 0.02
LDL-C (mmol/L)	0.43 ± 0.04	0.61 ± 0.03^a^	0.46 ± 0.04^b^	0.47 ± 0.02^b^

All values represent the means ± SEMs

Student’s unpaired *t*-test at W3 and ANOVA at W13 were performed. *n* = 6 in each group

*W3* week 3, *W13* week 13, *NL* normal litter, *SL* small litter, *CUR* curcumin, *TC* total cholesterol, *TG* triglyceride, *HDL-C* high-density lipoprotein cholesterol, *LDL-C* low-density lipoprotein cholesterol

^a^*p* < 0.05 versus NL

^b^*p* < 0.05 versus SL

### Energy expenditure

**Fig. 5:**
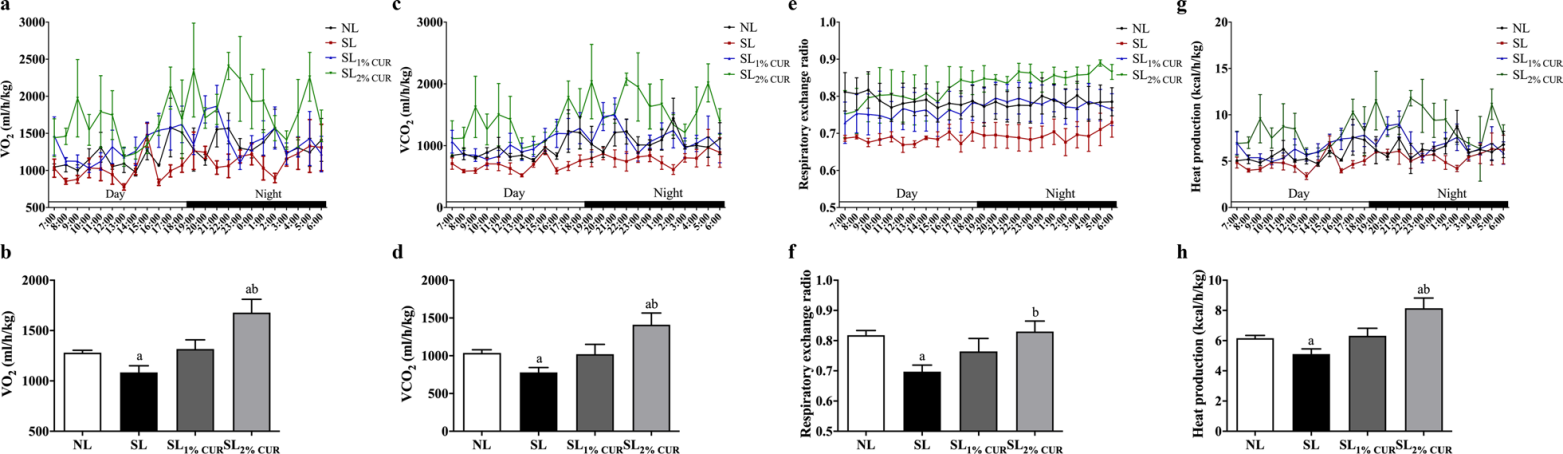
The whole-body metabolic rate, including oxygen consumption (**a** and **b**), carbon dioxide production (**c** and **d**), respiratory exchange ratio (**e** and **f**) and heat production (**g** and **h**) in rats at W13. All values represent means ± SEMs. ^a^*p* < 0.05 versus NL, ^b^*p* < 0.05 versus SL. ANOVA was performed. *n* = 4 in each group

We found that there were no obvious differences in the phenotypes mentioned above between NL rats and NL rats with dietary CUR supplementation (Additional file 1: Supplementary Figs. 2-5). These findings prompted us to form the hypothesis that CUR had no significant effect on postnatal normal feeding rats. Therefore, we next mainly explored the effects of CUR on energy expenditure and the browning of adipose tissue in postnatal overfeeding rats. At W13, the VO_2_ (Fig. 5a, b), VCO_2_ (Fig. 5c, d), RER (Fig. 5e, f), and heat production (Fig. 5g, h) of SL rats were all lower than those of NL rats. Compared with SL rats, the levels of VO_2_, VCO_2_, RER, and heat production were improved significantly in the SL_2% CUR_ rats but not in the SL_1% CUR_ rats. Similarly, the rectal temperature in SL_2% CUR_ rats was higher than that in SL rats at W13 (Additional file 1: Supplementary Fig. 6).

### Expression of browning-related genes in SAT

**Fig. 6:**
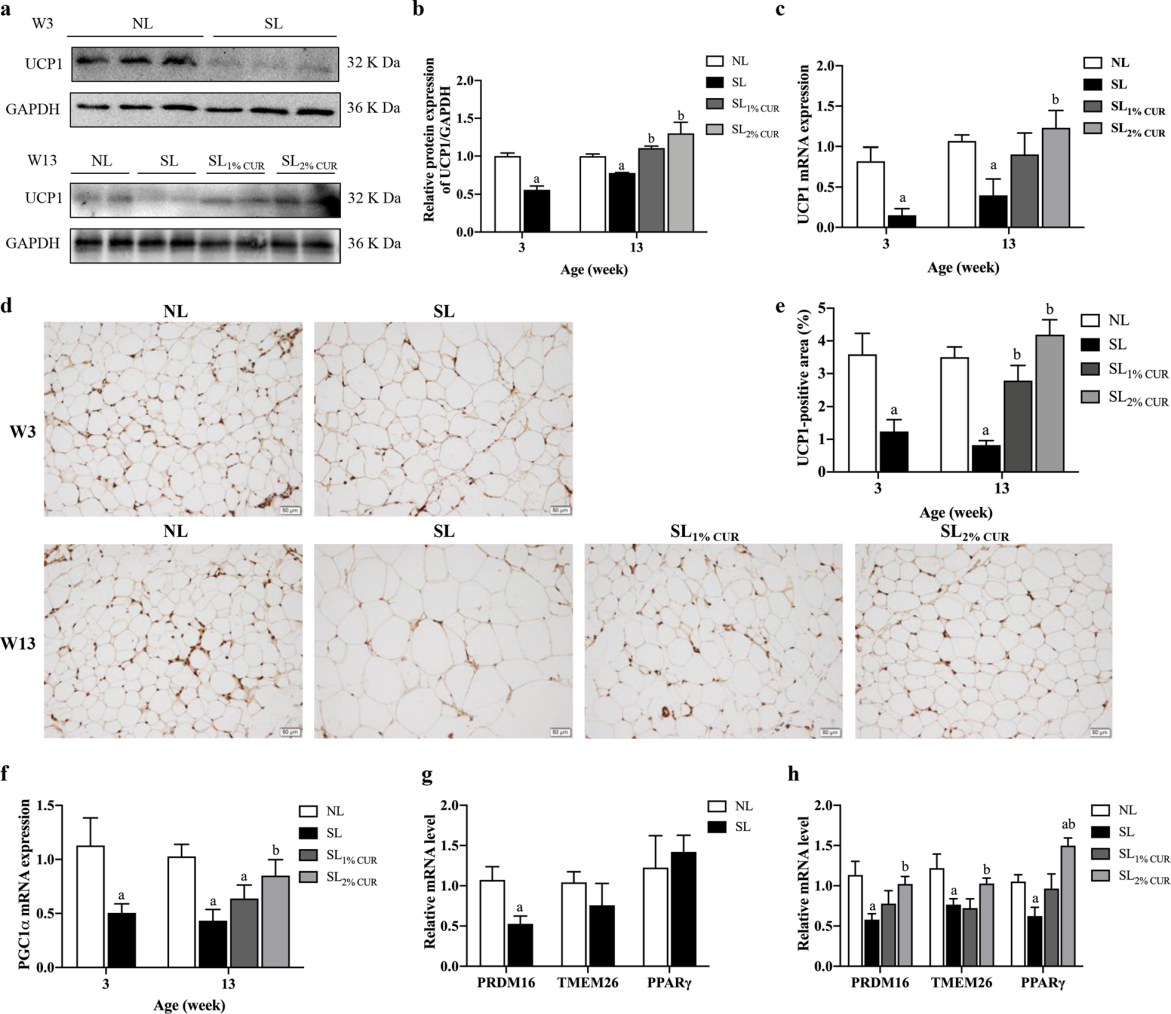
Protein (**a** and **b**) and mRNA (**c**) expression of UCP1, immunohistochemical analysis of UCP1 (**d** and **e**) and mRNA expression of PGC1α (**f**) in subcutaneous adipose tissue from rats at W3 and W13. mRNA expression of PRDM16, TMEM26 and PPARγ of subcutaneous adipose tissue in rats at W3 (**g**) and W13 (**h**). All values represent means ± SEMs. ^a^*p* < 0.05 versus NL, ^b^*p* < 0.05 versus SL. Statistical analysis was performed using Student’s unpaired *t*-test at W3 and ANOVA at W13. *n* = 6 in each group

To evaluate the effect of CUR on WAT browning, the expression levels of genes involved in the transformation of white adipocytes into beige adipocytes in SAT were detected. In comparison to NL, UCP1 mRNA and protein expression levels in SL rats were inhibited at W3 and W13 but were significantly up-regulated in SL_CUR_ rats. This trend became more obvious in the 2% CUR-supplemented rats at W13 (Fig. 6a–c). Consistently, the UCP1 protein-positive area in SAT decreased in SL rats at W3 and W13, and increased in SL rats with CUR supplementation (SL_1% CUR_ and SL_2% CUR_), compared to that in SL rats at W13 (Fig. 6d, e). Subsequently, the mRNA levels of PGC1α in SAT were consistent with those of UCP1 (Fig. 6f).

In addition, PRDM16 mRNA level was decreased in SL rats, whereas the expression levels of PPARγ revealed no difference between NL and SL rats at W3 (Fig. 6g). At W13, in comparison to NL rats, PRDM16 and PPARγ expression levels in SAT were suppressed in the SL rats and enhanced in the SL rats with dietary CUR supplementation, as expected. Likewise, the mRNA levels were enhanced more obviously in the SL_2% CUR_ rats (Fig. 6h). Moreover, TMEM26 mRNA levels of SAT in SL rats showed a decreasing trend at W3, till a significant difference at W13, which was upregulated in SL rats fed with a diet containing 2% CUR (Fig. 6g, h).

### Serum NE levels and β3-AR gene expression in SAT

**Fig. 7:**
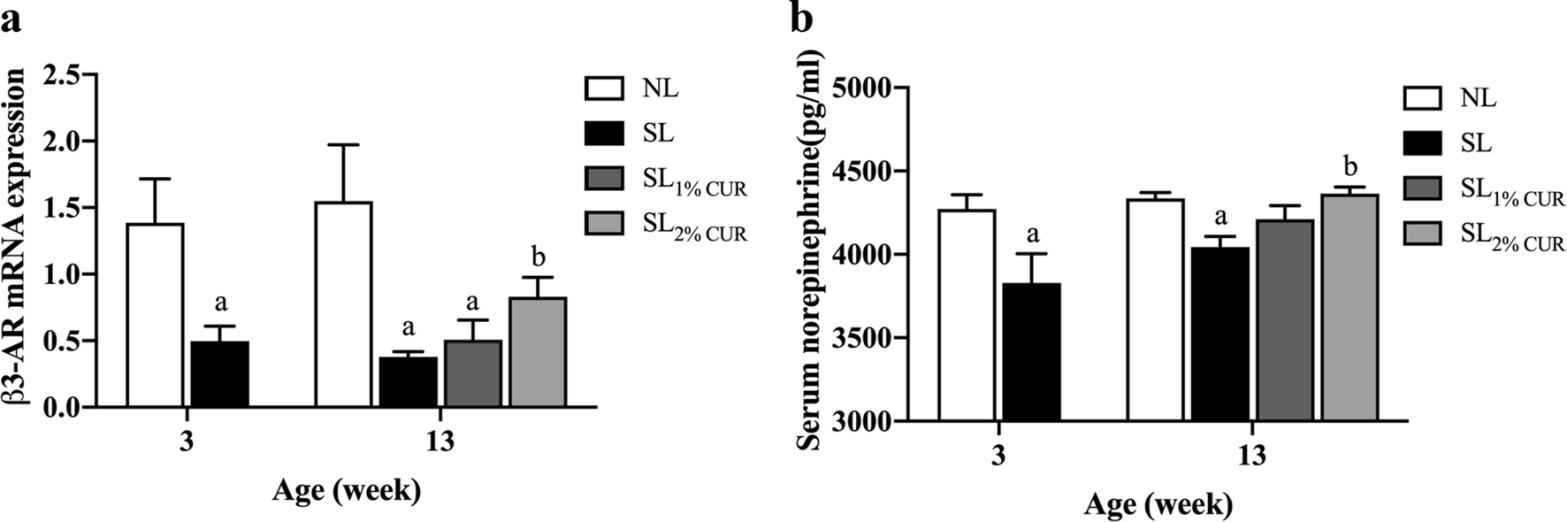
Serum levels of norepinephrine (**a**) and mRNA expression of β3-AR in subcutaneous adipose tissue (**b**) in rats at W3 and W13. All values represent means ± SEMs. ^a^*p* < 0.05 versus NL, ^b^*p* < 0.05 versus SL. Statistical analysis was performed using Student’s unpaired *t*-test at W3 and ANOVA at W13. *n* = 6 in each group

The thermogenic response mediated by UCP1 is regulated mainly by the sympathetic nervous system through releasing NE and ligating to β3-AR on the membrane of adipocytes. This study investigated whether CUR affected the release of NE together with its receptor expression. As illustrated in Fig. 7a, at W3 and W13, the serum NE level of SL rats was significantly lower than that of NL rats. Moreover, the serum NE reached a normal level only in the SL_2% CUR_ rats. The pattern of β3-AR mRNA expression in SAT among the groups was similar to that of serum NE (Fig. 7b).

## Discussion

Numerous studies on humans and rodents have proven that the suckling period nutritional environment could affect weight and energy homeostasis into adulthood [31, 32]. In this research, the postnatal overfeeding rat model, which is induced by SL rearing, resulted in several metabolic dysfunctions, including higher body weight and WAT mass, increased serum lipids and insulin resistance at weaning and adulthood. These results were in accordance with our previous studies [6, 33]. Rats fed a postweaning diet supplemented with CUR exhibited lower body weight, less fat mass, higher energy expenditure and improved glucolipid metabolism during adulthood compared to a standard diet in SL-reared rats. Furthermore, brown-like adipocytes with high UCP1 expression emerged in the SAT of these rats with CUR intervention. These data suggest that a dietary CUR supplement could stimulate the development of WAT browning and might be a strategy to increase energy expenditure to prevent obesity induced by postnatal overfeeding.

CUR’s capacity as an anti-obesity nutraceutical that increases weight loss and lowers fat mass has been verified in adulthood obesity models [34, 35]. This study is the first to exhibit this effect in overfed rats in an SL-rearing model. Recently, several important window phases from fetal formation to childhood have been identified, and studies have found that effective intervention during these phases could have a profound impact and may prevent the progression of obesity in adulthood [36]. This study proved that dietary CUR supplementation after weaning (SL_1% CUR_ and SL_2% CUR_ rats) could significantly reduce the body weight, adipose tissue mass, body fat percentage and adipocyte volume of rats. The size of adipocytes varies across different metabolic environments [37]. For example, overnutrition enlarges adipocytes to store excess fatty acids [38], while cold stimulation or physical exercise can induce lipolysis and increase the number of thermogenic beige adipocytes that are smaller than white adipocytes, thus reducing the adipocyte volume [11]. In the current study, we found more clusters of smaller UCP1-positive adipocytes in SAT of CUR-treated SL rats, suggesting that CUR decreases fat mass and the adipocyte volume through the conversion of white to beige adipocytes.

Many metabolic diseases arise from obesity in children and teens, including type 2 diabetes, insulin resistance and hyperlipidemia [39, 40]. This study initially found that dietary CUR supplementation could correct glucose intolerance, hyperinsulinemia and hyperlipidemia induced by postnatal overfeeding. Studies have shown that the size of adipocytes is related to the severity of obesity-linked diseases, such as diabetes and insulin resistance [41]. Smaller adipocytes have been proven to improve insulin sensitivity in WAT [42]. Here, we also detected smaller adipocytes and higher expression of genes regulating insulin sensitivity, including phosphoinositide 3-kinase (PI3K), protein kinase B (AKT1), AKT2, and glycogen synthase kinase-3 (GSK3b), in the SAT of SL rats with CUR supplementation (Additional file 1: Supplementary Fig. 7). In addition, a low-grade chronic inflammation in adipose, either systemic or local, plays a key role in the development of obesity and metabolic syndromes [43]. However, we observed no obvious differences in the mRNA levels of pro- and anti-inflammatory factors in SAT among groups at W13 (Data not shown).

Keeping energy intake and consumption in a balanced state is the basis for maintaining a healthy weight and metabolism. The rationales of treatments for weight loss usually include reducing total energy uptake and increasing energy expenditure [44]. In the present study, dietary administration of CUR (1% or 2% diet) raised energy expenditure but did not affect food intake in SL rats, which suggests that CUR could enhance energy metabolism rather than inhibit energy intake. Several studies have shown that CUR has the ability to upregulate the basal metabolic rate, which could lead to higher energy expenditure [45]. Unsurprisingly, this study observed an increase in the energy expenditure of SL rats fed a diet containing 2% CUR.

Adipose tissue, as a caloric reservoir, plays a critical role in regulating the balance of systemic energy metabolism [7]. Induction of WAT browning could increase energy consumption and help to alleviate metabolic disorders [9, 10, 46]. Upregulation of UCP1 expression is closely related to increased energy consumption and adaptive thermogenesis and is usually used as an indicator during BAT activation and WAT browning [47]. In rodents, WAT depots have different propensities to form beige adipocytes [48]. Induction of WAT browning occurs more easily in subcutaneous depots than in visceral mesenteric or epididymal depots [49, 50]. In this study, we mainly observed the browning feature of CUR in SAT and found that 2% dietary CUR supplementation could increase both UCP1 mRNA and protein expression in SAT in SL rats. PGC1α is a vital factor in UCP1 transcription [8]. Consistent with that of UCP1, the mRNA level of PGC1α in SAT increased in rats fed with 2% dietary CUR supplementation. Several molecular pathways are involved in browning. PRDM16 and PPARγ can gear PGC1α to increase the expression of UCP1 [51]. In the present study, 2% dietary CUR supplementation also increased PRDM16 and PPARγ mRNA levels in SAT. It is worth noting that PPARγ mRNA level maintained normal after W3, which may be attributed to a compensatory action to resist metabolic stress induced by postnatal overfeeding [52]. Moreover, transmembrane protein 26 (TMEM26) [8] is a specific beige-selective gene that can distinguish beige adipocytes from brown or white adipocytes in adipose tissues. Consistent to UCP1, the mRNA level of TMEM26 in SAT was increased in SL rats with 2% dietary CUR supplementation at W13. These findings suggested that CUR may act as a thermogenic activator to induce SAT browning, which could partly explain the benefits of dietary CUR intervention on metabolic disorders in obese rats resulting from postnatal overfeeding.

Furthermore, this study observed serum NE and β3-AR mRNA expression in the SAT of rats. The release of NE from the adrenal medulla and sympathetic terminals in WAT is mandatory for the immediate activation of existing beige adipocytes and the differentiation of beige adipocytes from their precursors [53]. NE binds to β3-AR expressed in brown/beige adipocytes and then activates c-AMP pathway-dependent mitochondrial UCP1, which is a critical mediator of adaptive thermogenesis in brown and beige adipose tissue [8]. The β3-AR signaling pathway is stimulated by several factors in WAT, the most effective is chronic cold exposure, which could induce the browning process [15]. In this study, we found that SL rats experienced a decrease in serum NE and β3-AR mRNA expression levels in their SAT at both W3 and W13, but this could be reversed by feeding them a 2% CUR dietary supplement postweaning. In vitro, our previous study found that browning marker gene expression was markedly upregulated following treatment with CUR in preadipocytes, and the increase was suppressed by a β3-AR antagonist [54]. Taken together, our findings support that CUR-induced SAT browning may be associated with sympathetic stimulation through the norepinephrine-β3-AR pathway.

The effects of functional diets are closely related to the dose administered [55]. Generally, orally ingested CUR is metabolized in the liver and small intestine, and liver enzymes (AST and ALT) are usually used to detect oral toxicity [56]. In this study, no significant difference was found in the serum AST and ALT in the SL_1% CUR_ or SL_2% CUR_ rats compared to the NL rats, which suggests that neither dosage of CUR adopted in the current research caused liver injury. In previous studies, the major intervention for CUR in obese animal models was oral administration, including gavage and dietary supplementation. The concentrations used in the latter method range widely, from 0.1% to 3%. In this study, we provided SL rats with two different doses of CUR (95% standardized CUR extract) to understand the effects of dose differences. The doses set in the current study were 1% and 2%, referring to the concentrations of dietary CUR (95% standardized CUR extract) used in previous studies on obese mouse models induced by a high-fat diet and Western diet, which were 1% and 3%, respectively. In the present study, the changes in body weight, serum chemical parameters, adipocyte surface area, and energy expenditure as well as the expression of browning-related genes in the SL_2% CUR_ rats were slightly better than those in the SL_1% CUR_ rats, but there were no significant differences in these obesity indicators and browning genes between the SL_1% CUR_ and SL_2% CUR_ rats. Therefore, dietary 2% CUR supplementation might be an ideal dose for treating postnatal overfeeding-induced obesity. However, whether a higher dose of CUR could lead to a stronger effect still needs further research.

## Limitation

In present study, we confirmed that dietary CUR supplementation is sufficient to promote browning of SAT and increase energy expenditure to attenuate obesity and related metabolic disorders induced by postnatal overfeeding. However, these effects should be validated in other organs, including liver, muscles, BAT, and other types of WAT. Moreover, we only found that CUR stimulated SAT browning by NE/β3-AR, and this mechanism should be testified in a knock-out rat model. Furthermore, multiple factors, such as extracellular vesicles and microbiota, may be involved in WAT browning [57, 58]. All these need further explorations.

## Conclusions

Browning program occurs mainly by sympathetic stimulation and interaction with norepinephrine (NE) with AR-β3, initiating a cascade of signal transduction that ends with the overexpression of UCP1 and other thermogenic proteins. Interestingly, a postweaning diet supplemented with CUR significantly reduced obesity and metabolic disorders in postnatal overfed rats induced by SL rearing, accompanied by the upregulated expression of UCP1and other browning-related genes in SAT. It is suggested that CUR is a potential “browning agent”. Importantly, CUR stimulated the browning program possibly regulated by NE/β3-AR. Based on these findings, we concluded that CUR extracted from natural and edible plants could be a new viable strategy to fight against postnatal overfeeding-induced obesity and related metabolic disorders.

## Supplementary Information

**Additional file 1.**. Supplementary Materials.

## Data Availability

Data are all contained within the article.

## Abbreviations

β3-AR: β3-Adrenergic receptor
AKT1: Protein kinase B
ALT: Alanine aminotransferase
ANOVA: Analysis of variance
AST: Aspartate aminotransferase
AUC: Area under the curve
BAT: Brown adipose tissue
CUR: Curcumin
EAT: Epididymal adipose tissue
GAPDH: Glyceraldehyde-3-phosphate dehydrogenase
GSK3b: Glycogen synthase kinase-3 beta
HDL-C: High-density lipoprotein cholesterol
HFD: High-fat diet
IPGTT: Intraperitoneal glucose tolerance test
LDL-C: Low-density lipoprotein cholesterol
LSD: Least significant difference
NE: Norepinephrine
NL: Normal litter
PGC1α: Peroxisome proliferator-activated receptor γ coactivator-1α
PI3K: Phosphoinositide 3-kinase
PPARγ: Peroxisome proliferator-activated receptor γ
PRDM16: Positive regulatory domain containing 16
RAT: Retroperitoneal adipose tissue
RER: Respiratory exchange ratio
SAT: Subcutaneous adipose tissue
SL: Small litter
TC: Total cholesterol
TG: Triglyceride
TMEM26: Transmembrane protein 26
UCP1: Uncoupling protein 1
VCO2: Carbon dioxide production
VO2: Oxygen consumption
WAT: White adipose tissue
W3: Postnatal week 3
W10: Postnatal week 10
W13: Postnatal week 13
