# Supplementary material for: Dietary curcumin supplementation promotes browning and energy expenditure in postnatal overfed rats
Source: Nutr Metab (Lond). 2021 Oct 30;18:97. doi: 10.1186/s12986-021-00625-5 (PMC8557570; doi:10.1186/s12986-021-00625-5)
Supplement: Supplementary file 1 — Additional file 1.. Supplementary Materials. [file 12986_2021_625_MOESM1_ESM.docx]

**Supplementary Materials**

**Dietary curcumin** **supplementation promotes browning and energy expenditure in** **postnatal overfed rats**

Xiaolei Zhu, Susu Du, Qinhui Yan, Cuiting Min, Nan Zhou, Wei Zhou, Xiaonan Li

**Supplementary Method**

**Temperature**

Rectal temperature was determined by inserting a lubricated rectal temperature probe (MC-347, OMRON, Japan) about 2 cm into the rectum of the rat at W3 and W13, respectively. The measurement was carried out for 4 consecutive days at fixed time point (8:00 am to 9:00 am), the first 3 days were the adaptation period, and the result of the 4th day was used as the final rat rectal temperature data.

**Supplementary Table**

Primer sequences used for supplementary mRNA quantification by RT-qPCR

|  | Forward primer 5’-3’ | Reverse primer 5’-3’ |
| --- | --- | --- |
| PI3K | ATGCAACTGCCTTGCACATT | CGCCTGAAGCTGAGCAACAT |
| AKT1 | GCCCAACACCTTCATCATCC | GTCTCCTCCTCCTGCCGTTT |
| AKT2 | CACAGAGAGCCGAGTCCTACA | GGCATACTCCATCACAAAGCA |
| GSK3b | CTTTTCACAGGGCTACGC | ACAGGCAAGCACATTTCC |

PI3K Phosphoinositide 3-kinase, AKT Protein kinase B, GSK3b Glycogen synthase kinase-3 beta.

**Supplementary Figures**


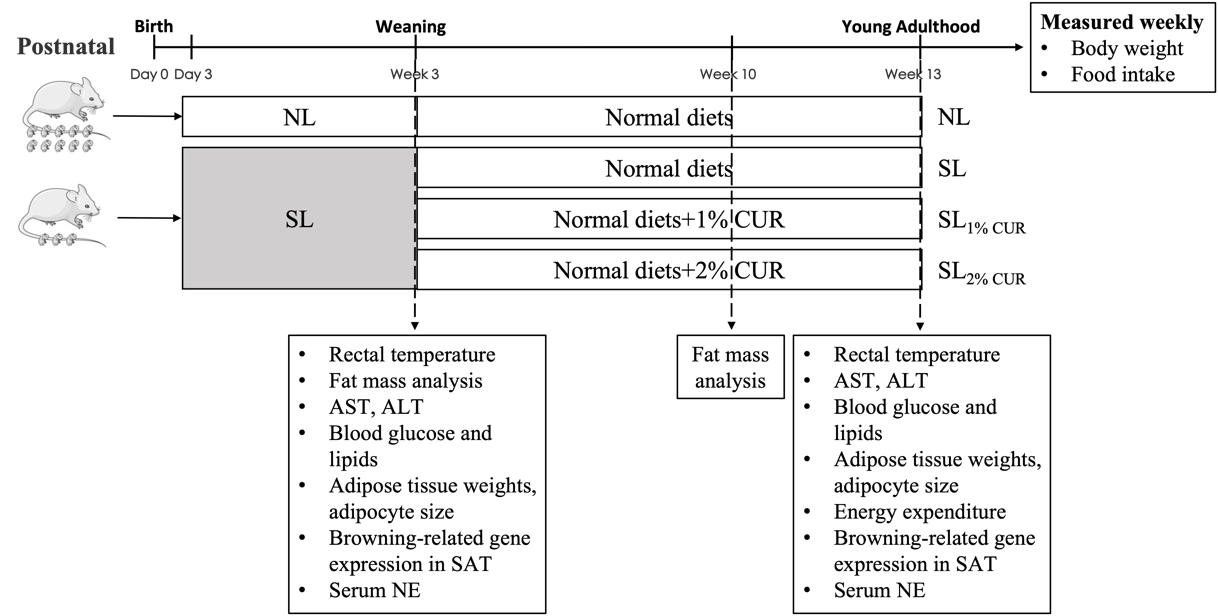


**Supplementary Fig. 1.** **Schematic diagram of the design of the experiment.**


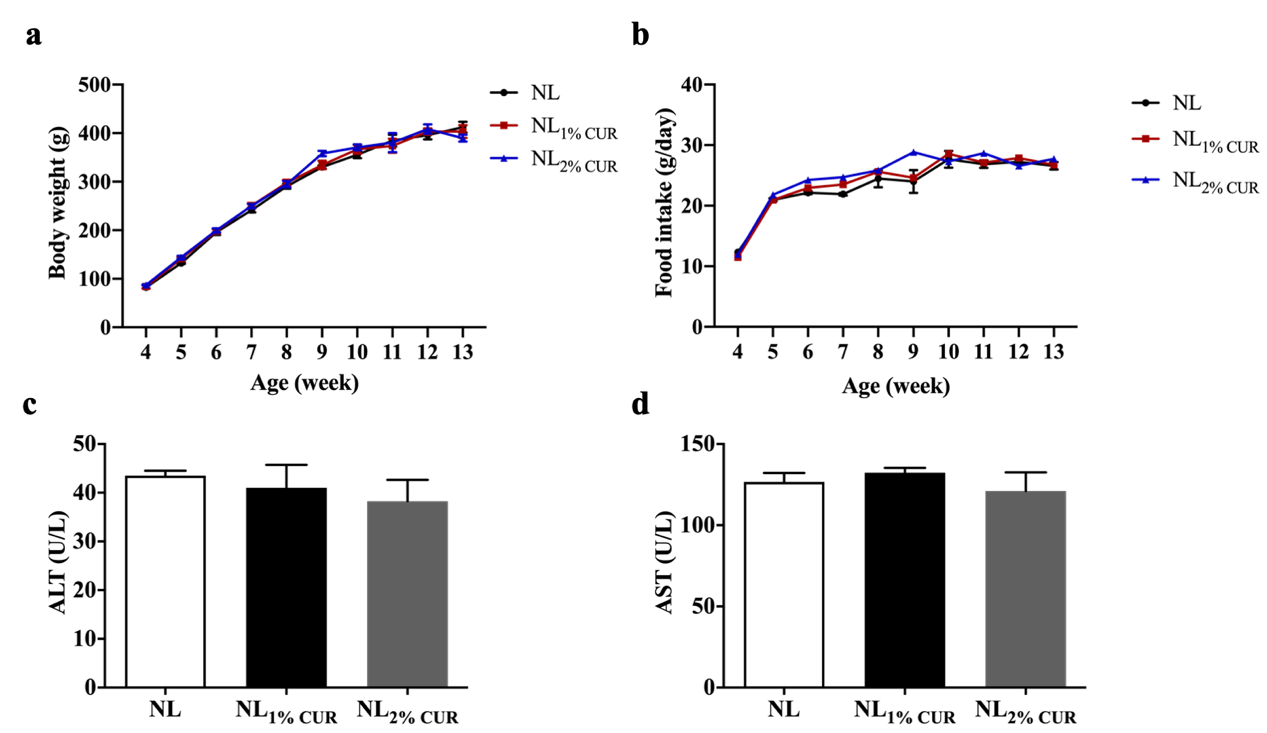


**Supplementary Fig. 2. Effects of dietary curcumin supplementation on body weight, food intake and liver enzymes in postnatal normal feeding rats.** Body weight (a) and food intake (b) in rats from week 4 to week 13. Serum levels of alanine aminotransferase (ALT) (c) and aspartate aminotransferase (AST) (d) in rats at week 13. All values represent means ± SEMs. Data were analyzed by one-way analysis of variance (ANOVA). *n*=6 in NL group, *n*=3 in NL_1% CUR_ and NL_2% CUR_ group. NL Normal litter, CUR Curcumin, ALT Alanine aminotransferase, AST Aspartate aminotransferase.


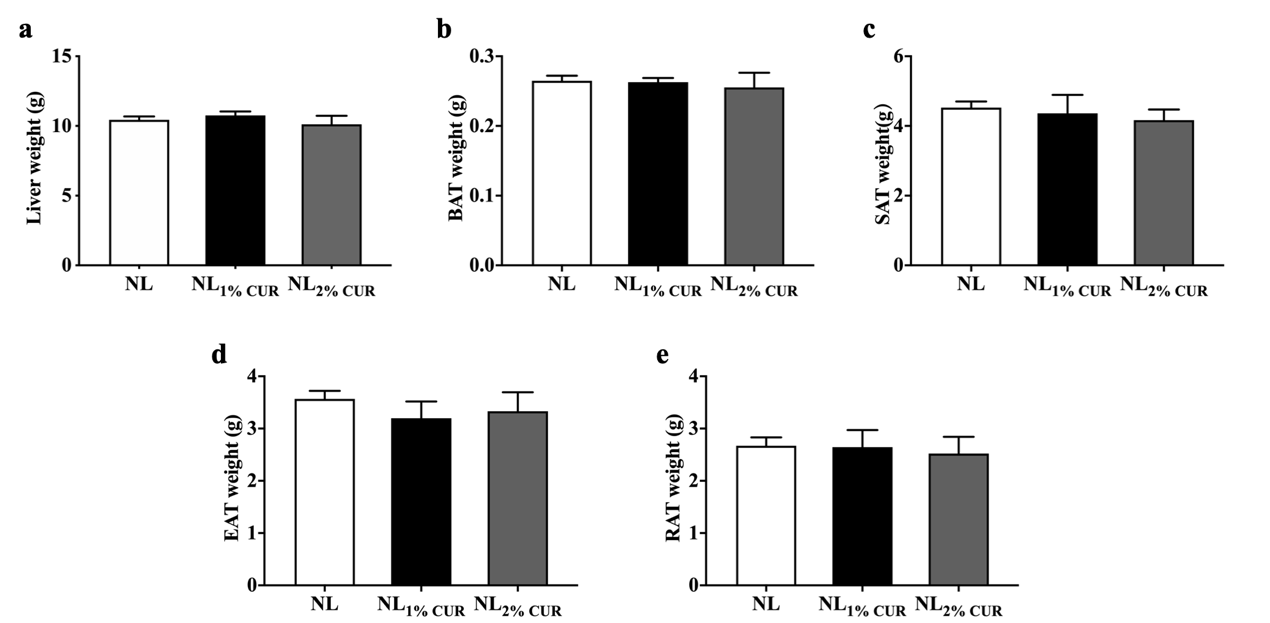


**Supplementary Fig. 3.** **Effects of dietary curcumin supplementation on liver and adipose tissue weights in postnatal normal feeding rats.** Liver (a), BAT (b), SAT (c), EAT (d) and RAT (e) weights in rats at Week 13. All values represent means ± SEMs. Data were analyzed by one-way analysis of variance (ANOVA). *n*=6 in NL group, *n*=3 in NL_1% CUR_ and NL_2% CUR_ group. NL Normal litter, CUR Curcumin, BAT Brown adipose tissue, SAT Subcutaneous adipose tissue, EAT Epididymal adipose tissue, RAT Retroperitoneal adipose tissue.


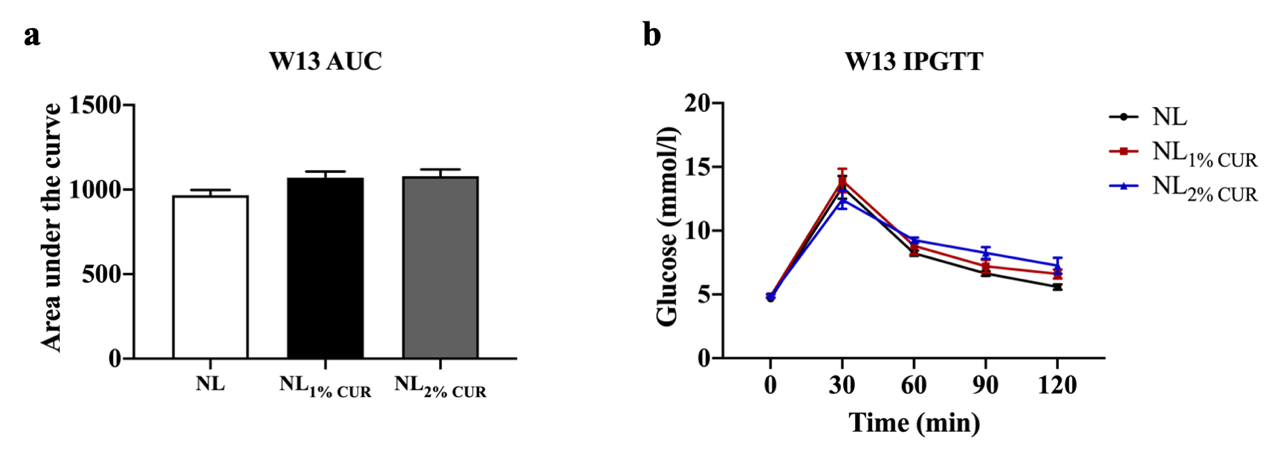


**Supplementary Fig. 4. Effects of dietary curcumin supplementation on glucose homeostasis in postnatal normal feeding rats.** IPGTT (a) and AUC (b) at week 13. All values represent means ± SEMs. Data were analyzed by one-way analysis of variance (ANOVA). *n*=6 in NL group, *n*=3 in NL_1% CUR_ and NL_2% CUR_ group. NL Normal litter, CUR Curcumin, IPGTT Intraperitoneal glucose tolerance test, AUC Area under the curve.


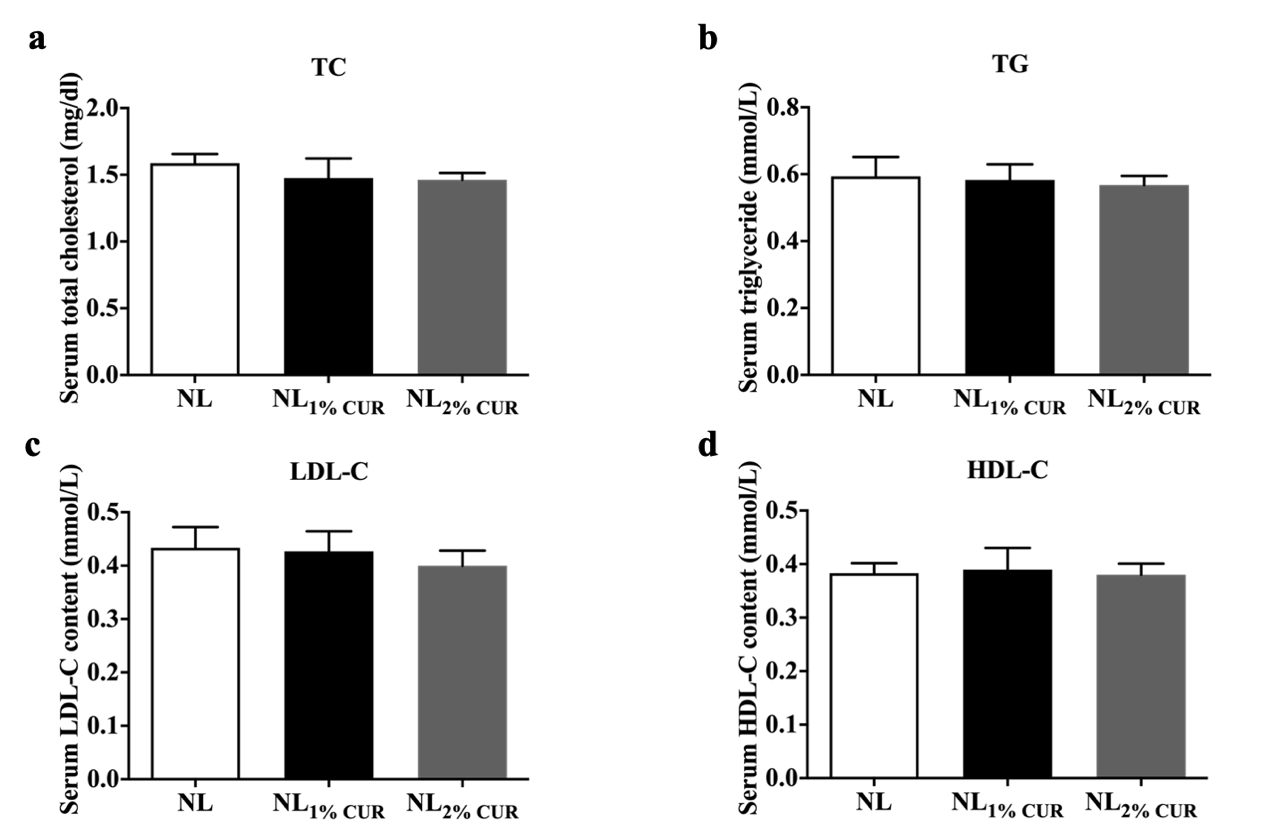


**Supplementary Fig. 5. Effects of dietary curcumin supplementation on** **serum lipids** **in postnatal normal feeding rats.** Serum TC (a), TG (b), LDL-C (c) and HDL-C (d) levels in rats at week 13. All values represent means ± SEMs. Data were analyzed by one-way analysis of variance (ANOVA). *n*=6 in NL group, *n*=3 in NL_1% CUR_ and NL_2% CUR_ group. NL Normal litter, CUR Curcumin, TC Total cholesterol, TG Triglyceride, HDL-C High-density lipoprotein cholesterol, LDL-C Low-density lipoprotein cholesterol.


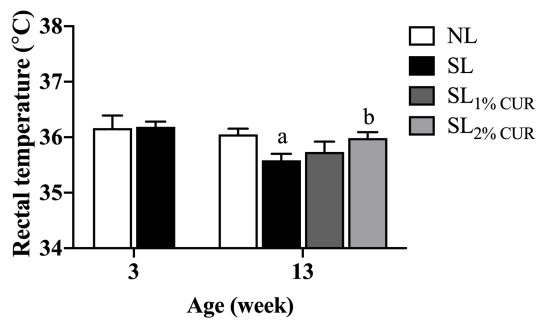


**Supplementary Fig. 6.** **Rectal temperature of rats at W3 and W13.** All values represent means ± SEMs. ^a^*p* < 0.05 versus NL, ^b^*p* < 0.05 versus SL. Statistical analysis was performed using Student’s unpaired *t*-test at W3 and ANOVA at W13. *n*=6 in each group.


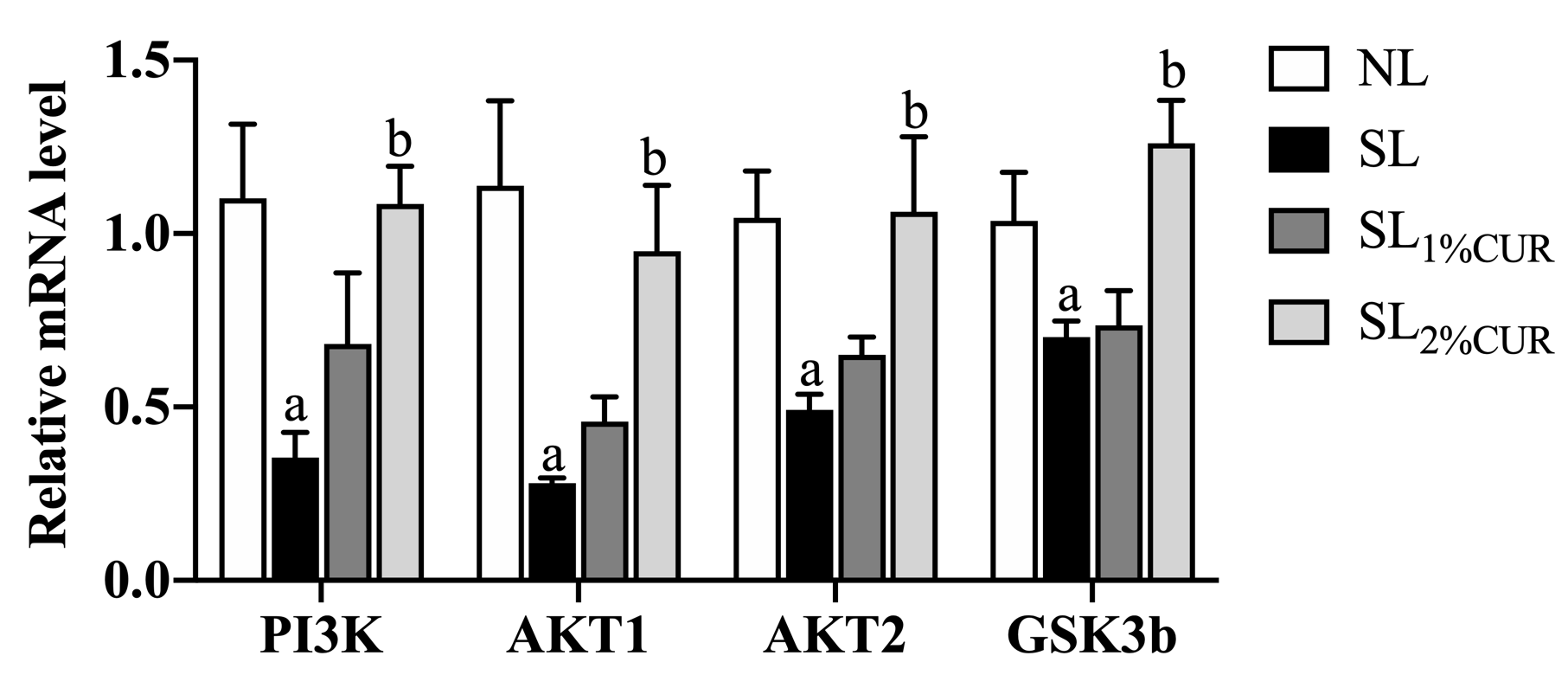


**Supplementary Fig. 7. Effects of dietary curcumin supplementation on mRNA levels of insulin signaling pathway in SAT in postnatal overfeeding rats.** All values represent means ± SEMs. Data were analyzed by one-way analysis of variance (ANOVA). NL Normal litter, SL Small litter, CUR Curcumin, PI3K Phosphoinositide 3-kinase, AKT Protein kinase B, GSK3b Glycogen synthase kinase-3 beta. *n*=6 in each group.
